# Na/K Pump and Beyond: Na/K-ATPase as a Modulator of Apoptosis and Autophagy

**DOI:** 10.3390/molecules22040578

**Published:** 2017-04-21

**Authors:** Cassiano Felippe Gonçalves-de-Albuquerque, Adriana Ribeiro Silva, Camila Ignácio da Silva, Hugo Caire Castro-Faria-Neto, Patrícia Burth

**Affiliations:** 1 Laboratório de Imunofarmacologia, Instituto Oswaldo Cruz, FIOCRUZ, Rio de Janeiro RJ CEP 21040-900, Brazil; cassianofg@gmail.com (C.F.G.-d.-A.); arsilva71@gmail.com (A.R.S.); hugocfneto@gmail.com (H.C.C.-F.-N.); 2 Laboratorio de Imunofarmacologia, Departamento de Bioquímica, Universidade Federal do Estado do Rio de Janeiro, Rio de Janeiro RJ CEP 20211-010, Brazil; 3 Laboratório de Enzimologia e Sinalização Celular, Departamento de Biologia Celular e Molecular, Instituto de Biologia, Universidade Federal Fluminense, Niterói RJ CEP 24020-141, Brazil; camila_ignacio@id.uff.br

**Keywords:** Na/K-ATPase, non-small cell lung cancer, cardiotonic steroids, apoptosis, autophagy

## Abstract

Lung cancer is a leading cause of global cancer deaths. Na/K-ATPase has been studied as a target for cancer treatment. Cardiotonic steroids (CS) trigger intracellular signalling upon binding to Na/K-ATPase. Normal lung and tumour cells frequently express different pump isoforms. Thus, Na/K-ATPase is a powerful target for lung cancer treatment. Drugs targeting Na/K-ATPase may induce apoptosis and autophagy in transformed cells. We argue that Na/K-ATPase has a role as a potential target in chemotherapy in lung cancer treatment. We discuss the effects of Na/K-ATPase ligands and molecular pathways inducing deleterious effects on lung cancer cells, especially those leading to apoptosis and autophagy.

## 1. Introduction

Many new cancer treatments have arisen, but the search for a suitable drug that acts on cancers that are chemo-resistant to common cancer drugs remains a huge challenge. Lung cancer is one of the most common cancers and remains a leading cause of global cancer deaths. The highly invasive phenotype, rapid progression, and resistance to chemotherapy of lung cancer contribute to its poor prognosis [1]. In 1957, when Skou described Na/K-ATPase and its primary function, he probably could not imagine that years later this pump would be shown to play a role in cell signalling and be considered a target for cancer treatment. Epidemiological data indicate that samples of breast cancer tissue from patients with congestive heart failure treated with cardiac glycosides exhibit more benign features than tissue samples from control cancer patients who were not treated with cardiac glycosides [2]. A recent systematic review, however, reported a 34% increase in breast cancer risk with the use of cardiac glycosides, but it is unclear whether this association reflects a confounding or causal relationship [3]. The anticancer effects of cardiac glycosides have also been examined in cancers other than breast cancer, including leukaemia and tumours of the kidney/urinary tract [4]. In addition, the use of digoxin can prevent prostate cancer [4].

Cardiotonic steroids (CS), when bound to Na/K-ATPase, trigger several cell-signalling pathways, resulting in the proliferation, differentiation and promotion of autophagy or apoptosis. These effects vary depending on the cell type as well as the type and concentration of CS. In addition, through enzyme inhibition, CS may also elicit changes in the response of kinases to changes in ATP and calcium. Interestingly, normal and tumour cells express different pump isoforms, and Na/K-ATPase acts as a CS receptor. Thus, the pump is a powerful target for antitumor molecules, and molecules targeting Na/K-ATPase are currently being tested in clinical trials.

Na/K-ATPase has been studied as a target for cancer treatment. CS trigger intracellular signalling upon binding to Na/K-ATPase. Thus, Na/K-ATPase is a powerful target for lung cancer treatment. We argue that Na/K-ATPase has a role as a potential target in chemotherapy in lung cancer treatment. Here, we discuss the effects of Na/K-ATPase ligands and the molecular pathways inducing deleterious effects on lung cancer cells, especially those leading to apoptosis and autophagy.

## 2. Lung Cancer

Lung cancer is the major cause of cancer deaths around the world. In the USA, it is estimated that lung cancer was responsible for approximately 27% of cancer deaths in 2016 [5]. The number of Americans who die each year from lung cancer is approximately equal to the number of annual deaths due to prostate, breast, and colon cancer combined [6]. There are two main types of lung cancer: non-small and small cell lung carcinomas, which are pathologically and clinically distinct. The most common is non-small cell lung cancer (NSCLC), which represents more than 80% of cases [7,8].

NSCLC is commonly subdivided into three subtypes: squamous cell carcinoma (SQCC), large cell carcinoma (LCLC) and adenocarcinoma. Adenocarcinoma is the most common subtype, representing more than 40% of NSCLC cases. In 2013, a new classification of only two types of NSCLC was proposed: adenocarcinoma and neuroendocrine tumour, abolishing the LCLC subtype [9].

A high cancer rate of adenocarcinoma has been described in both sexes, especially in women, and is associated with lifestyle [5,10,11]. Studies have shown that the greatest cause of adenocarcinoma is not smoking but ethnicity and genetic susceptibility. Smoking is usually associated with small cell lung cancer (SCLC) and squamous-cell carcinoma [12].

NSCLC is a heterogeneous cancer with cellular and genetic diversity. Molecular genotyping of several samples of adenocarcinoma has revealed targets for therapy, and the detection of these alterations could also be predictive markers for therapeutic success. Mutations in epidermal growth factor receptor (EGFR) or rearrangements in the anaplastic lymphoma kinase (ALK) gene are the most common findings—almost 25% of adenocarcinoma cases. These alterations generally cause increased activity of EGF and ALK. However, the most observed change in NSCLC is mutation of the tumour suppressor TP53, which is not a treatment target. Mutations in other tumour suppressor genes such as Liver Kinase B1/Serine Threonine Kinase 11 (LKB1/STK11), neurofibromatosis type 1 (NF1), cyclin-dependent kinase Inhibitor 2A (CDKN2A), SMARCA4 and kelch-like enoyl-CoA hydratase associated protein (KEAP) that cause loss of function also occur [13]. The heterogeneity of mutations makes treatment difficult due to the frequent occurrence of resistance. The high mortality rate is generally related to late diagnosis and acquisition of chemo-resistance [14]. Until a few years ago, treatment essentially comprised chemotherapy and drugs combined with platinum, with a small improvement in quality of life and a high mortality rate. New therapies have emerged based on inhibitors of EGFR, such as erlotinib, gefitinib, or afatinib. Approximately 25%–30% of NSCLC have Kirsten rat sarcoma viral oncogene homolog (KRAS) mutations, mostly adenocarcinomas. However, no drug targeting KRAS has been developed [15].

Clinical treatment has also been based on minor genetic alterations in NSCLC tumours, such as in ROS1, RET, and MET [15,16,17,18]. Acquired resistance to chemotherapy remains a challenge in the development of an effective treatment. In this context, Na/K-ATPase has emerged as an attractive target for anticancer therapy.

## 3. Na/K-ATPase

**Figure 1:**
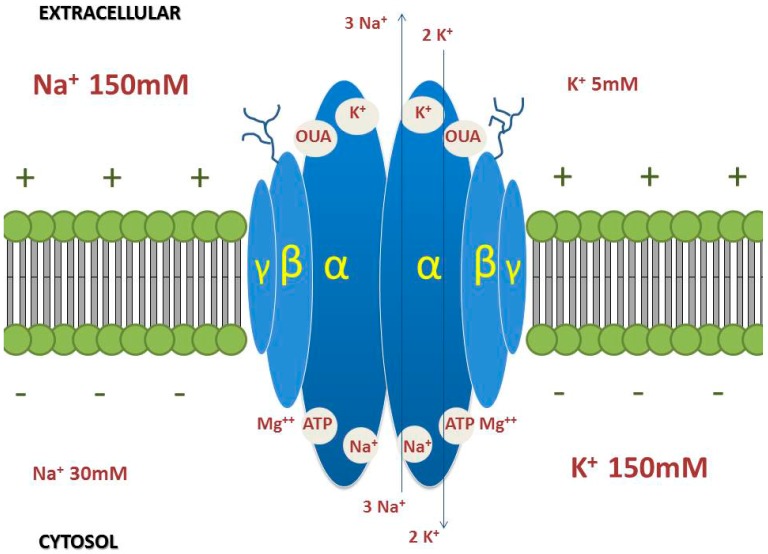
Scheme of the insertion of Na/K-ATPase into the plasma membrane. Ionic transport is accomplished by ATP hydrolysis and also depends on the physiological concentrations of the ions inside and outside the cell. The α-subunits (with sites for Na, K, ATP, and cardiac glycosides: OUA (ouabain)), β-subunits (glycoprotein) and γ subunits are shown.

Na/K-ATPase is a heteromeric protein complex located on the plasma membrane of eukaryotic cells that uses ATP to transport sodium ions out of the cell and potassium ions into cells [19,20,21,22,23]. Na/K-ATPase is a member of the P-type ATPase class, with intermediate participation of the pump in the phosphorylated form. During phosphorylation, the protein undergoes a conformational transition from helix to beta sheet [24]. This transition results in the phosphorylation of the enzyme by ATP in the presence of Mg^2+^ and Na^+^ ions and dephosphorylation in the presence of K^+^ ions [19,25]. Na/K-ATPase functions in cellular electrochemical gradient maintenance, osmotic balance, conductivity in nerves and muscles, cell adhesion and motility [23,26,27], and triggering of intracellular signalling [28]. Na/K-ATPase is composed of three subunits, α-subunit, β-subunit and γ-subunit. The α-subunit has four isoforms (α1, α2, α3 and α4), the beta subunit has three isoforms (β1, β2 and β3), and the gamma subunit has seven isoforms (FXYD1–7) [29,30]. The combination of isoforms most common in tissues is α1β1; the diverse distribution of different subunits in organs and tissues has been reviewed in Mijatovic et al. 2012 [31] (Figure 1).

### 3.1. α-Subunit

The α-subunit consists of 10 transmembrane helices (M1–M10) with a total molecular mass of 110 kD. The *N*- and *C*-termini are located in the cytosol, and the majority of the segments are located in the intracellular space [32,33]. The binding sites for ATP and Mg^2+^, cardiac glycosides as well as Na^+^ and K^+^ ions are all located in the α-subunit, which is considered the catalytic subunit [19,20]. The four α-subunit isoforms are expressed differently in different tissues throughout the development of the organism. The α1-isoform is expressed in several cell types and is predominant in the kidneys and liver. Importantly, the α1-subunit is up-regulated in certain cancer types, including NSCLC [34], renal cell carcinoma [35], glioma [36] and melanoma [37]. The α2-isoform is present mostly in the brain, heart muscle, and skeletal muscle. The α3-isoform, by contrast, is found in the central nervous system, cardiac muscle, skeletal muscle and placental tissue, whereas the α4-isoform is restricted to the testes [29,33,38].

### 3.2. β-Subunit

The β-subunit is formed by a transmembrane segment with a molecular weight of 55 kD. The *N*-terminus is located in the cytoplasm, whereas the *C*-terminus (glycosylated) is located in the extracellular medium [33]. This glycoprotein is considered regulatory and is involved in the stabilization of the enzymatic complex in the plasma membrane, the affinity of the pump for potassium and sodium ions, and adhesion processes through *E*-cadherin [39,40,41,42]. Three β-subunit isoforms (β1, β2 and β3) have been identified. Isoforms β1 and β2 are present predominantly in mammalian cells. The β1-isoform is distributed throughout all tissues, whereas the β2-isoform is concentrated in nervous tissue, heart, cartilage and erythrocytes. The β3-isoform is found predominantly in nerve tissue, as well as in skeletal muscle and the lung [29,33,34].

### 3.3. γ-Subunit

**Figure 2:**
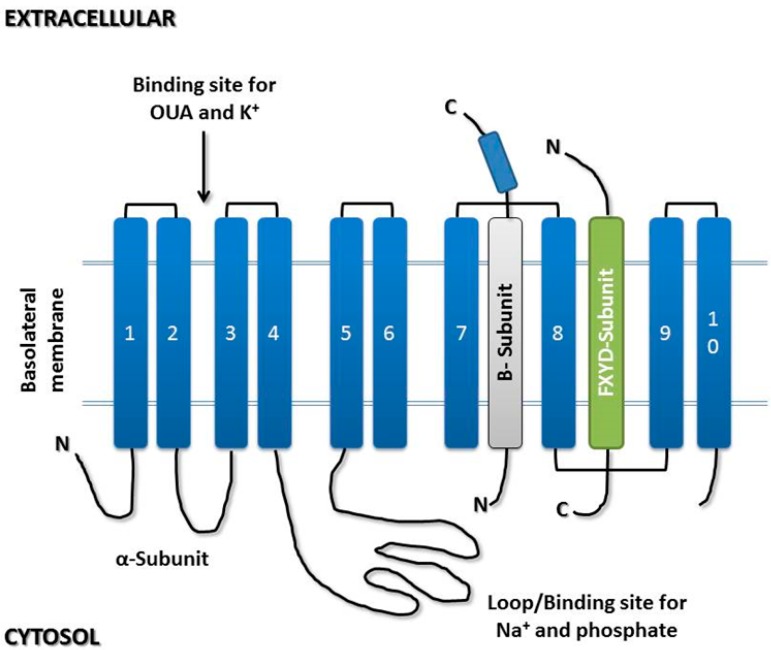
Schematic representation of the subunit domains of Na/K-ATPase, which is composed of a catalytic α-subunit (blue), a glycosylated β-subunit (grey), and, in some tissues, a single transmembrane span containing an extracellular invariant FXYD sequence (green).

The γ-subunit has only one transmembrane domain and a molecular mass of 15 kD. Seven isoforms of the γ-subunit have been described (FXYD1–7) [29,30,42]. The γ-subunit is a proteolipid present in different tissues such as kidney tissues, cardiac tissues and skeletal muscle and belongs to the FXYD family. The seven isoforms differ in the amino acid residues in the *N*-terminal domain [29,42]. Its function seems to be associated with the modulation of the enzyme affinity for different ligands, with a direct and positive effect on the maximum rate of adenosine triphosphate (ATP) hydrolysis, and thus the γ-subunit is considered regulatory in addition to the β-subunit (Figure 2) [43,44].

## 4. Na/K-ATPase: Expression in Cancer and Potential of Cardiotonic Steroids

Several changes in the expression of Na/K-ATPase have been observed in cancer cells, such as elevation of activity during the transformation of malignant cells. Roles in cell survival, proliferation, adhesion and migration have also been described [45,46,47]. In this context, variations in the expression of the different subunits of the enzyme compared to normal tissues have been described, including in renal carcinoma cells, NSCLC, and glioma, where there is an elevation of α1-isoform expression. In colon carcinoma, there is an elevation of the α3-isoform, and in prostate carcinoma, there is a decrease in α1-isoform expression (reviewed in [31]).

Although the distribution of the different isoforms under pathological conditions such as cancer is not completely clear, the α-subunit is considered a target for new anticancer therapies. In general, in tumours, the α1-isoform is highly expressed in the early stages of tumorigenesis, with low expression in later stages in favour of an elevation of α3-isoform expression [42,48,49]. Thus, the pump may have a role not only as a biological marker but also as a therapeutic target for cancer. In this context, studies have evaluated the therapeutic potential of Na/K-ATPase modulators, such as cardiotonic steroids. Different molecules such as ouabain, exhibit activity against numerous types of cancer cells acting on the induction of apoptosis, cell cycle arrest or even autophagy [42,50,51].

## 5. Cardiotonic Steroids

Cardiac glycosides or cardiotonic steroids (CS) are a group of compounds isolated from plants and animals. The main structural feature of CS is the central steroid nucleus, in which an unsaturated lactone ring replaces the D-ring at C-17. CS are classified as cardenolides and bufadienolides depending on the nature of the lactone. The most well-known CS are the cardenolides digoxin, digitoxin, ouabain and oleandrin and the bufadienolides bufalin and hellebrin. These CS have been used for years in the treatment of heart failure and arrhythmia [34,52,53]. Na/K-ATPase inhibition causes an elevation of intracellular sodium, activating the Na^+^/Ca^2+^-exchanger and consequently resulting in increased intracellular calcium and increased cardiac muscle contractility [54]. Endogenously produced CS have been detected in the blood, adrenal gland, and hypothalamus but do not inhibit Na/K-ATPase; these endogenous CS are associated with cardiovascular and renal disease [55,56]. The stability of the CS complex with a Na/K-ATPase and the inhibitor potency depends on the stereochemistry of the sugar [20,38]. Most cardenolides show higher affinity for the α2- and α3-isoforms [20]. Bufadienolides, however, have a higher affinity for the α1-isoform.

The effects of CS in cancer cells include but are not limited to apoptosis sensitization and anoikis, affecting chemo-resistant cells as well [45,57].

Thus, CS have been considered excellent anticancer substances, even at very low concentrations. Drugs such as huachansu, digoxin, and Anvirzel are in phase II trials as anticancer agents for various types of cancer, including NSCLC. They may represent an alternative for tumours resistant to the usual chemotherapeutic agents. Several mechanisms of CS-mediated anticancer activity were postulated by Mijatovic et al. in 2008 [58]. The two classes of CS and their mechanisms of action are briefly discussed below.

### 5.1. Cardenolides

Ouabain, one of the most studied CS, was isolated from the plant *Strophanthus gratus*. Like other CS, it inhibits the Na/K-ATPase. The binding site for ouabain is located in the extracellular portion of the pump in the α subunit [59] and is the same for other CS such as digoxin [20] (Figure 1).

Analysis of Na/K-ATPase from shark rectal gland crystallized with K^+^ and ouabain revealed the interaction of an arginine residue (R887) with the rhamnose moiety of ouabain [60]. The R887 resides within the L7/8 loop of the shark α-subunit and is homologous to R886 of the rat α-subunit. Mutation of D885 and D886 to arginine residues greatly reduces ouabain binding ability [61], suggesting that the L7/8 loop is directly involved in the binding of CS [62].

Ouabain-like substances that act as hormonal signals in response to endogenous or exogenous stimuli such as stress, hypertension or even sports have been identified [53]. As an anticancer substance, ouabain has been shown to inhibit proliferation and cell migration and is capable of sensitizing chemo-resistant cells [16,63]. Ouabain presents great potential in neuroblastomas and is indicated for use in conjunction with chemotherapeutic agents for its ability to suppress tumour or cell growth by quiescence [64].

Extracted from the *Nerium oleander* plant, oleandrin is considered less toxic than but as potent as ouabain and digoxin [38]. As an inhibitor of Na/K-ATPase, oleandrin has higher affinity for the α3-isoform than the α1-isoform [65].

Digitoxin is a cardiotonic steroid isolated from the flower of *Digitalis purpurea*. Along with digoxin, digitoxin is one of the most used drugs for the treatment of heart failure and has a more stable profile [38]. Digoxin binds the α subunit in the αM4 domain and has higher affinity for the α2- and α3-isoforms [20]. Digitoxin, similar to digoxin and other cardiotonic steroids, has a strong antitumor effect, inhibiting the growth and proliferation of these cells as well as inducing apoptosis [66,67].

In a phase II trial, Kayali et al. 2011 showed that digoxin with erlotinib does not act in synergism on EGFR, indicating the need to identify more specific targets and chemotherapeutic combinations that increase the potential of digoxin in patients with NSCLC [68].

In addition to their potential in anticancer therapy, lung cancer, pancreatic cancer, and breast cancer, the cardiotoxicity of digitoxin and other cardenolides should be considered [69]. The concentrations of CS used in anticancer therapy and cardiovascular disease treatment have been investigated. At toxic doses, Na/K-ATPase inhibition is elevated (greater than 60%), causing Na^+^ and Ca^2+^ levels to increase and resulting in cardiac arrhythmia [70]. Consequently, therapeutic CS concentrations are restricted, and there is a search to reduce these toxic effects, such as UNBS1450 [71] (a hemi-synthetic derivative of 2′′-oxovoruscharin with trans-trans-cis steroid rings), in which molecular changes resulted in a decrease in cardiotoxicity and greater inhibitory capacity compared to classic CS [34].

### 5.2. Bufadienolides

The name bufadienolide is derived from the toad genus *Bufo*, which produces this class of substances used for many years in Chinese medicine for the treatment of cardiac disorders [72]. CS, as well as bufalin, exhibit anticancer activity mediated by the induction of apoptosis and autophagy in several tumours, such as glioma and hepatocellular carcinoma [73,74].

Hellebrin is a bufadienolide extracted from the plant *Helleborus niger* that exhibits greater affinity to the α1-isoform. It exhibits antitumor potential against cells that present MDR (multiple drug resistance) [75].

Bufadienolides exhibit antiproliferative behaviour in several human cancer cell lines by inducing death and cell cycle arrest, indicating its potential for use in anticancer therapy with effects similar to those of cardenolides.

**Figure 3:**
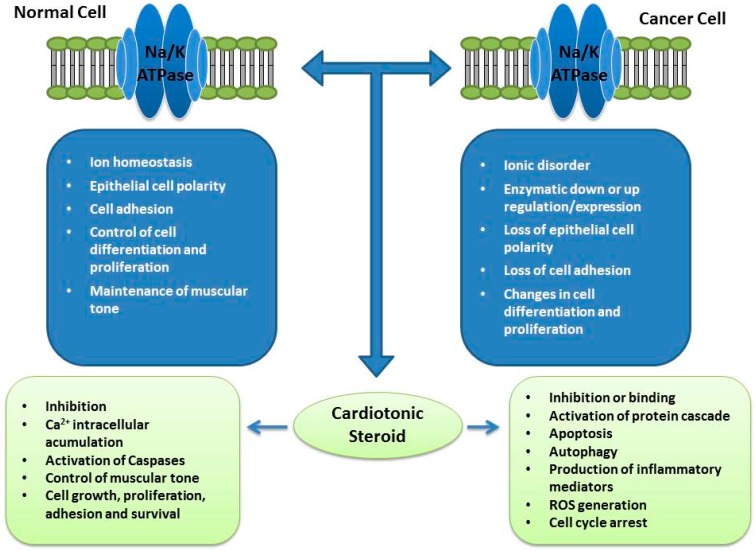
Functions of Na/K-ATPase enzymes in normal and cancer cells and their interaction with cardiotonic steroids. In normal cells, this pump is responsible for several functions, such as maintenance of ion homeostasis; maintenance of epithelial cell polarity; participation in the process of cell adhesion; control of cell differentiation and proliferation and maintenance of muscular tone. Its interaction with cardiotonic steroids results in enzymatic inhibition; Ca^2+^ intracellular accumulation; activation of caspases; control of muscle tone; cell growth, proliferation, adhesion and survival via signalling pathways. In cancer cells, there are several changes in Na/K-ATPase that result in ionic disorder; enzymatic down- or up-regulation of expression; loss of epithelial cell polarity and cell adhesion; changes in cell differentiation and proliferation. Interaction with cardiotonic steroids may result in inhibition; activation of protein cascade; apoptosis; autophagy; production of inflammatory mediators; reactive oxygen species (ROS) generation and cell cycle arrest. Most of these phenomena are linked to intracellular signalling mediated by the enzyme.

Figure 3 summarizes the functions of Na/K-ATPase and the effects of CS on normal and cancer cells (Figure 3).

## 6. Role of Na/K-ATPase in the Lung 

**Figure 4:**
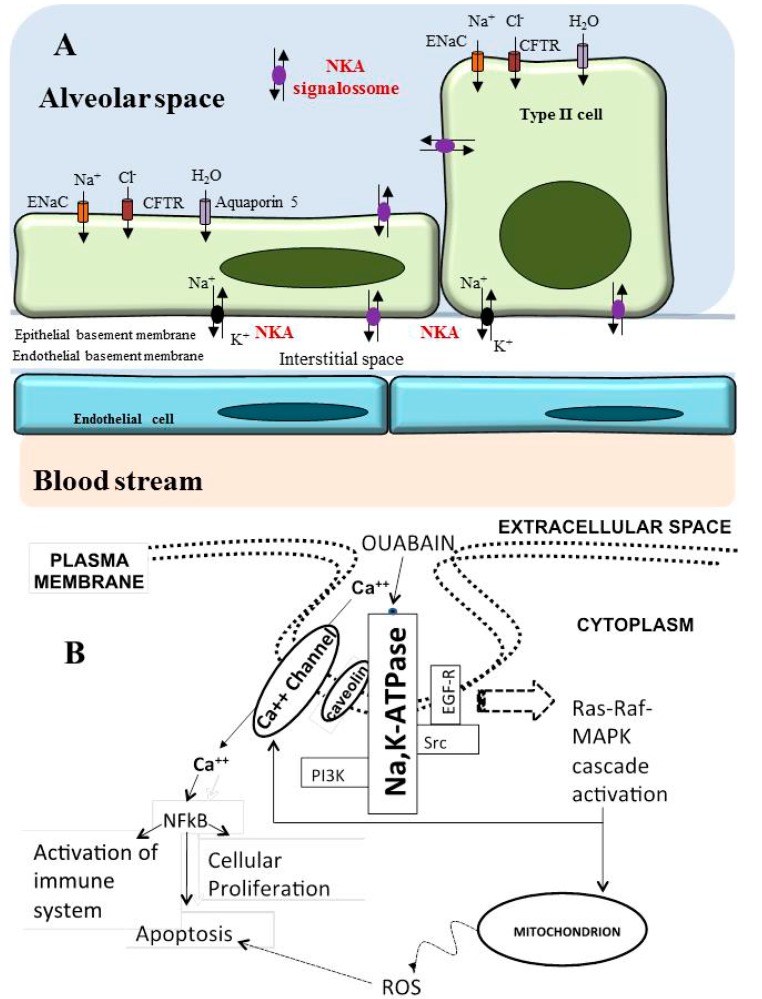
Structure of the alveolar–capillary barrier in the intact lung and Na/K-ATPase signalosome. Alveolar type I and II cells form the alveolar barrier and present the Na^+^ channel, Na/K-ATPase (NKA) and aquaporin 5. Endothelial cells form the capillary wall (**A**); Signalosome of Na/K-ATPase (**B**); Binding of cardiotonic steroids to Na/K-ATPase triggers a cascade of events starting with activation and phosphorylation of Src and caveolin-1, which leads to the transactivation of the epidermal growth factor receptor (EGFR). Activation of the Ras-Raf-MAPK cascade increases cytoplasmic Ca^2+^ and activates the production of reactive oxygen species (ROS) by the mitochondria. Augmented Ca^2+^ activates NFκB, leading to immune system activation, cellular proliferation or apoptosis. Other recruited proteins include PLC (not shown) and PI3K. The downstream effects are various and include inhibition of the cytoprotective effects of NF-kB and Akt and the activation of AP-1 and Erk1/2, leading eventually to cell death via apoptosis and autophagy. However, the type of response depends on the cell type, glycoside concentration and exposure time. ENaC—Epithelial sodium channel; CFTR—Cystic fibrosis transmembrane conductance regulator; EGFR—Epidermal growth factor receptor; NFκB—Nuclear factor kappa-light-chain-enhancer of activated B cells; PLC—Phospholipase C; PI3K—Phosphoinositide 3-kinase; Akt—Protein kinase B; AP-1—Activator protein 1; Erk—Extracellular signal-regulated kinases.

Basolaterally located Na/K-ATPase together with the apically located epithelial sodium channel (ENaC) in epithelial lung cells are responsible for alveolar fluid clearance (AFC). AFC is driven by sodium transport across the airway epithelium, with removal of water from the alveoli to the bloodstream. Chloride transport via the cystic fibrosis transmembrane conductance regulator (CFTR) is also important for AFC [76]. Water crosses the alveolar epithelium either paracellularly via tight junctions or transcellularly via aquaporins (AQP). AQP5-deficient mice (Figure 4A) have significantly decreased airway-capillary water permeability [77]. Endogenous acetylcholine increases AFC via the activation of alveolar epithelial Na/K-ATPase [78] (Figure 4A).

Impairment of the enzyme Na/K-ATPase during acute respiratory distress syndrome (ARDS) not only prevents resolution of lung oedema but also intensifies its formation. Thus, sodium transport and oedema clearance are associated with better outcomes in patients with sepsis and ARDS [76]. Ouabain inhibits AFC in isolated, perfused fluid-filled mouse lungs [79] and in animals in vivo [80,81,82,83,84]. The overexpression of FXYD1 in the lungs of ARDS patients may limit Na/K-ATPase activity and contribute to oedema persistence [85]. Therefore, molecules targeting Na/K-ATPase have toxic effects on the heart and likely in the lungs as well because of the narrow therapeutic window (0.5–0.9 ng/mL for digoxin) [86]; these molecules should therefore be used with caution. For instance, renal disease prolongs the half-life of digoxin, with elevation of its serum concentration [87]. This elevation causes an array of clinical side effects such as nausea and vomiting, visual disturbances and disorientation, and arrhythmia [86]. Mutations and fluctuations in the Na/K-ATPase are directly linked to certain mechanisms in several diseases, including diabetes mellitus and Alzheimer’s disease. Alterations in the enzyme may increase cancer cell growth. The cellular processes and pathways influenced by these effects include but are not limited to MAPK, PI3K, Akt/mTOR, and epigenetic methylation, as reviewed by Durlacher et al. 2015 [88,89]. Pump internalization in response to stimuli also alters its function. Hypercapnia leads to JNK-induced phosphorylation of LMO7b, a scaffolding protein, which promotes the endocytosis of Na/K-ATPase in alveolar epithelial cells [89].

Diverse proteins interact with Na/K-ATPase and trigger downstream events. The α-subunit of Na/K-ATPase serves as an anchoring platform for protein–protein interactions [90].

Low concentrations of CS may generate a cellular signal by two different mechanisms. The first mechanism involves the so-called plasmERosome. The α2- or α3-isoform of the pump in a specific plasma membrane raft located near the membranes of the sarcoplasmic/endoplasmic reticulum (SR/ER) is inhibited in smooth muscle cells, hippocampal neurons, or astrocytes of rodents [91]. This inhibition of the α2- or α3-isoform results in a transient increase in the sub-plasmalemmal sodium concentration, which activates NCX1 and leads to a small, local increase in calcium concentration. This is followed by the release of Ca^2+^ from the SR/ER, which triggers an intracellular signalling cascade, including the induction of smooth muscle cell contraction, which is postulated to be one of the causes of essential hypertension [92].

In the second mechanistic model, Na/K-ATPase is part of a caveolae-defined environment of proteins called the signalosome (Figure 4B). Na/K-ATPase is associated with a complex of proteins in the caveolae that transmits different signals to the intracellular environment [93] and acts as a receptor for CS. Mutational analysis showed that the caveolin binding motif of the α-subunit of Na/K-ATPase is required for the translocation of both the α-subunit of Na/K-ATPase and Na^+^/H^+^-exchanger to the basolateral membrane [94]. The Na/K-ATPase signalosome provides survival signals in normal cells when CS bind to the sodium pump [28,93,95,96] but death signals in cancer cells [31,37,58,85,97,98,99,100,101].

The signalosome may trigger the activation of the inositol trisphosphate receptor (IP3R), PI3K, Src/caveolin and focal adhesion kinase (FAK) [28,91,95,102,103]. The activation of Na/K-ATPase localized within the signalosome may induce conformational changes in the pump, which are transduced to the IP3R of the SR/ER [103,104]. The PI3K/Akt/mTOR pathway regulates cell proliferation, apoptosis, and autophagy [105,106]. Activation of IP3R, Src-dependent phosphorylation and activation of phospholipase C (PLC)-α1 stimulate IP3 formation and Ca^2+^ release from the SR/ER, ultimately resulting in protein kinase C (PKC) activation [107,108,109,110]. Src kinase activation triggers the phosphorylation of the EGFR and the activation of the Ras/Raf/MEK/Erk 1/2 pathway [111]. Ouabain inhibits Na/K-ATPase and activates p38-MAPK [112] and NFκB, eventually promoting apoptosis. Pump inhibition suppresses Src through a tyrosine residue located on the α-subunit of the pump [72,113]. Src kinase activity is regulated by both ATP and ADP concentrations [88], and contrary to the initial hypothesis, there is no solid evidence for a direct molecular interaction between Na/K-ATPase and Src under physiological conditions [89]. Activation of these signalling cascades not only stimulates gene activation and cell proliferation but also provides a synergistic effect between the calcium-dependent effects of CS and the calcium-independent signalling events that occur upon the interaction of CS with Na/K-ATPase.

The Ras/Raf/MAPK signalling cascade is also activated when CS interact with non-pumping sodium pump mutants [114], indicating that a local sodium concentration elevation followed by a rise in calcium concentration is not necessarily required for the induction of the signalling process [91]. Curiously, CS induce endothelial nitric oxide synthase (eNOS) phosphorylation and NO production in human umbilical vein endothelial cells, and this effect is associated with CS-induced Na/K-ATPase activation at low (nM) concentrations [115].

Na/K-ATPase down-regulation has been described in multi-drug resistant (MDR) and P-glycoprotein-overexpressing cells, and the downstream signalling pathways are also deregulated. Na/K-ATPase signalosome deregulation seems to favour the MDR phenotype [116]. As recently reported, CS, at low concentrations, activate the Src-EGFR-Ras-Raf-kinase pathway through Na/K-ATPase, inhibiting the proliferation and survival of tumour cells [48]. Stress conditions in the hypothalamus–pituitary–adrenal axis lead to the exhaustion of adrenal production of endogenous digitalis-like compounds, which may contribute to tumorigenesis in chronic stress situations. In those cases, low production (pM) will predominantly stimulate the proliferation of tumour cells [117].

### Na/K-ATPase as a Modulator of Apoptosis and Autophagy in Non-Small Cell Lung Cancer

The ability of CS to evade cancer cell resistance is related to the differential regulation of gene expression in cancer cells. The increased sensitivity of cancer cells to CS compared to normal cells can be explained by many molecular mechanisms, involving intracellular signalling proteins and differential regulation of transcription [45,88,118].

Examples describing the involvement of intracellular signalling proteins and mutations in different types of cell death caused by CS and related Na/K-ATPase effects on the signalosome as targets are described below.

The ability of CS to induce cell death includes enhanced radiosensitivity of H460 and A549. CS have no effect on H1299, a p53 null cell, indicating that the effects of CS are dependent on p53. P53 is an oncogene widely implicated in DNA repair and apoptosis. CS contained in huachansu suppress the cell viability of cancer cells of the p53 phenotype and increase levels of caspase-3 and cleaved poly-(ADP-ribose) polymerase (PARP). Huachansu also decreases the expression of Bcl-2 and p53, reinforcing apoptosis as the cell death mechanism involved in the effect of CS. HCS increases radiation sensitivity via p53- and caspase-3-dependent mechanisms rather than apoptosis and impairment of DNA repair [119].

Bufalin inhibits A549 proliferation and Akt activation and synergizes with Akt inhibitors to induce apoptosis and increase Bax expression. Those phenomena support a role of bufalin in negatively modulating NSCLC proliferation and apoptosis, which involves an increase in Bax expression, a decrease in Bcl-2 and livin expression, and caspase activation, all characteristic components of programmed cell death [120].

TNF-related apoptosis-inducing ligand (TRAIL) resistance often causes lung cancer progression. Ouabain induces apoptosis of H292 cells in response to TRAIL and increases caspase-3 activation and PARP cleavage at concentrations non-toxic to normal cells. Ouabain also causes ROS production and down-regulation of anti-apoptotic Mcl-1. ROS trigger Mcl-1 degradation in the proteasome. Ouabain induces the expression and activation of pro-apoptotic proteins and the degradation of anti-apoptotic proteins [121].

Sensitivity to TRAIL can be increased via post-transcriptional mechanisms, such as by death receptor 5 mRNA stabilization in NSCLC. Na/K-ATPase ligands trigger HuR translocation, which stabilizes death receptor 5 mRNA, a mechanism that could account for the ability of CS to counteract TRAIL tolerance and induce apoptosis [122]. 

Mutation of STK11 is also associated with lung cancer progression. Digoxin and ouabain exhibit selective effects on STK11 mutant cancer cell lines. Regular STK11 function reduces CS efficacy. Digoxin reduces the growth of STK11 mutant but not wild-type xenografts. In vitro experiments have confirmed these data, as digoxin induces ROS generation and G2/M arrest only in STK11 mutant A549 cells. Preventing ROS formation by *N*-acetyl-l-cysteine treatment reduces G2/M arrest caused by digoxin in STK11 mutant A549 cells. SiAMPK treatment of STK11 wild-type cells increases sensitivity to digoxin. Disruption of STK11-AMPK signalling is important for the effects of digoxin as a growth inhibitor [123].

Intracellular signalling disruption by Na/K-ATPase inhibitors leads to mitochondrial apoptosis. Perillyl alcohol is a Na/K-ATPase inhibitor [122]. Temozolomide-perillyl alcohol (TMZ-POH) exhibits potent cytotoxic activity against NSCLC, inhibiting cell proliferation by inducing G2/M arrest and DNA damage, decreasing the mitochondrial transmembrane potential and ROS accumulation, and activating MAP kinases, ultimately leading to apoptosis. The cytotoxicity of TMZ-POH is reversed by two ROS scavengers, catalase and *N*-acetyl-l-cysteine [124].

The pro-apoptotic effect of CS may also involve the extrinsic pathway, as demonstrated by studies using TXA9, a CS from *Streptocaulon juventas*. TXA9 induces cell cycle arrest in Sub G0/1 and G2/M periods. TXA9 activates the extrinsic pathway by inducing a signalling complex composed of Fas, FADD and pro-caspase 8, leading to the production of caspase-8, which activates caspase-3, an effector caspase, as an apoptosis inducer. In vivo analysis demonstrated that TXA9 efficiently reduces the tumour growth of NSCLS xenografts [125]. 

In silico analyses support the involvement of Na/K-ATPase in the effects of CS on apoptosis. In addition, co-immunoprecipitation and immunolocalization have shown a physical Na/K-ATPase-Bcl-2 protein interaction. A Na/K-ATPase antibody co-immunoprecipitates BCLXL and BAK protein in FHLC and A549 cells, confirming Na/K-ATPase-Bcl-2 complex formation [126].

The cytotoxic effect of digitoxin in NSCLC H1975 cells is caused by G2 phase arrest in cells resistant to tyrosine kinase inhibitors. Digitoxin overcomes tyrosine kinase inhibitor resistance and decreases cell growth via a mechanism dependent on α-tubulin expression and disruption of microtubule polymerization [127].

Defects in microtubule polymerization cause cell cycle arrest, detachment, and apoptosis. NSCLC H460 cells are sensitive to apoptosis caused by detachment, a mechanism called anoikis. Decreased Mcl-1 expression by proteasome degradation can cause NSCLC anoikis. A monosaccharide digoxin derivative can sensitize NSCLC to anoikis with higher potency than digoxin itself. This pro-apoptotic effect was observed only in cancer cells and not in normal small epithelial cells [128].

In a detailed analysis, Wang et al. 2012, showed that CS can induce G2/M arrest even at concentrations that do not cause apoptosis. Autophagy was observed, with increases in mean times and dose-dependent autophagic phenotype markers such as LC3-II, Atg5 and Beclin 1 [66]. Autophagy is a type of cell death followed by vacuolization of the cytoplasm [128]. 

AMPK signalling has also been reported to occur via mTOR deactivation. ERK 1/2 are also activated. Pharmacological tools targeting the autophagy cascade or siRNA block autophagy and enhance cellular viability, relating autophagy to tumour suppression. Digoxin and ouabain trigger autophagy-dependent growth inhibition involving AMPK-induced mTOR signalling down-regulation and ERK 1/2 activation [66].

Tat-Beclin 1 induces cell death by autophagy [129], a process called autosis [129,130]. Na/K-ATPase subunit knockdown inhibits autosis induced by Tat-Beclin 1 or starvation. Na/K-ATPase inhibitors exhibit similar effects [129].

Ouabain also induces cell death in a caspase-independent manner. Conversion of LC3-I to LC3-II is observed, revealing the presence of autophagic flux. Class III PI3k blocks ouabain-induced cell death, and reduction of Bcl-2 is observed in ouabain-treated cells. C-Jun *N*-terminal kinase (JNK) appears to participate in the ouabain-induced cascade because either pharmacological treatment with a JNK inhibitor or shRNA prevent Bcl-2 decrease, conversion of LC3-I and cell death. The molecular mechanisms involved in autophagy caused by ouabain include a decrease in Bcl-2 and consequent disruption of the interaction with Beclin 1 [129,131].

Src, MEK 1/2 and ERK 1/2 are indeed targets of ouabain and digoxin in A549 and H460 cells. These enzymes are activated after treatment and participate in the occurrence of autophagic phenotypes, as indicated by the disruption of MEK 1/2 and ERK activation and autophagy by the Src inhibitor PP2. The biochemical mechanism of ouabain or digoxin-induced autophagy also involves ROS generation. Src knockdown abolishes all of these phenomena in NSCLC cells, indicating a central role of Src in CS-induced autophagy [132].

Considering the involvement of Na/K-ATPase in numerous cellular functions, it is clear that changes in the expression and activity of this enzyme may be related to the pathophysiology of many diseases, making this pump a powerful therapeutic target [133]. Drugs targeting Na/K-ATPase are suitable candidates for cancer treatment because these molecules also target apoptosis-resistant and MDR cancer cells [45,51,134,135]. MDR cells exhibit down-regulation of Na/K-ATPase and deregulated downstream signalling pathways, but CS are able to evade MDR [116]. CS have a narrow therapeutic window because of their cardiovascular risk, which includes arrhythmia, and thus their use requires caution. The dose–response and dose–toxicity relationships of cardiac CS as anticancer drugs should be tested in preclinical studies to better design a clinical trial with proper randomization, controls and sample size. Such studies may provide further support for the use of CS as an anticancer agent in lung treatment.
